# The ‘availability’ bias: underappreciated but with major potential implications

**DOI:** 10.1186/cc13763

**Published:** 2014-03-12

**Authors:** Wassim H Fares

**Affiliations:** 1 Department of Internal Medicine, Section of Pulmonary, Critical Care, and Sleep Medicine, School of Medicine, Yale University, 15 York Street, LCI 105-C, New Haven, CT 06510, USA

## Abstract

Many biases have been described that potentially introduce prejudice or a systemic error into a study that would favor one outcome versus another. One major source of bias has, so far, been underappreciated: the availability bias. When the study intervention is available to clinicians outside of the clinical trial, the trial could become biased to favor the control study arm. Clinicians may, consciously or unconsciously, use this intervention outside of the trial on patients whom they believe would benefit from the intervention, and enroll in the trial those patients for whom they do not feel strongly about the benefit of the intervention. The clinicians do not always share the equipoise of the study investigators. This could have major implications on the analysis of clinical trials, including the systematic reviews that originate from such trials.

## 

Hundreds of biases in biomedical research have been identified [1]. However, a common bias of huge clinical implications is typically underappreciated and usually not well addressed. An intervention in a clinical trial that is already in the market and available to the potential recruiting health-care providers outside of the clinical trial may and would bias the enrolled study population toward a subgroup of patients who are unlikely to benefit from that intervention. Many past and ongoing trials investigate interventions that are already in practice. A prominent example is the trials of pulmonary artery catheter (PAC) in critically ill patients [2]. Other possible examples include trials of hypothermia [3], high-frequency oscillation [4], and recombinant activated protein C [5]. Even when this availability bias does not alter the overall results or conclusion of such studies, it could have an important impact on decreasing the overall amplitude of any potential effect signal. It usually moves the effectiveness results toward the null hypothesis and thus could tip the risk/benefit ratio more toward higher relative risk.

To elaborate on such an availability bias, it is worth exploring in more detail one of the above-mentioned interventions: the PAC, also known as the Swan-Ganz catheter in honor of the two scientists who designed it decades ago [6]. Multiple studies have been carried out since it was first introduced into the commercial market. It is unfortunate that this catheter became available in the market long before prospective clinical trials to document its efficacy were carried out, leading to decades of debate about its clinical utility. Clinicians are often faced with the dilemma of providing care with the tools available to them (including such catheters) versus agreeing to enroll their patients in a clinical trial in which they might not receive the intervention (if they are randomly assigned to the control arm of the study). Equipoise may be strongly valid according to the study investigators, but clinicians whose patients are needed for such trials may not share this equipoise.

Many of the patients screened for such trials are excluded because they already have the intervention as decided clinically by their health-care provider. In the Acute Respiratory Distress Syndrome Clinical Trials Network (ARDSnet) Fluid and Catheter Treatment Trial (FACTT), a multi-center, nationwide, elaborately designed trial sponsored by the National Institutes of Health’s National Heart, Lung and Blood Institute, the effect of placing a PAC in patients with acute respiratory distress syndrome (ARDS) was studied [7]. FACTT results suggested that there is no role for such catheters in managing patients with ARDS. The availability bias may have potentially played a significant role in altering the results of this trial. Although 513 patients were randomly assigned to the intervention arm of FACTT to receive a PAC, 20.8% of a total of 11,511 patients screened (that is, approximately 2,394 patients) were excluded from the trial because they had a PAC placed already. In addition, 15.9% (approximately 1,830 patients) were excluded because their clinicians declined to enroll their patients in this trial. Including hundreds of critically ill patients in a clinical trial is a great achievement and is not easy by any measure, and the ARDSnet should be applauded for such a great effort. The question remains whether or not the other thousands of excluded patients were the ones who would have benefited from such an intervention. Despite multiple randomized controlled trials, such as FACTT, and subsequent meta-analyses [2], it is not clear which subgroup of patients, if any, would actually benefit from such an intervention or any other intervention that was studied in a randomized controlled clinical trial in a setting in which the study intervention was available to patients and clinicians outside of the trial.

A more recent example includes high-frequency oscillation in early ARDS [4]. Seventy patients were excluded because they were already undergoing high-frequency oscillation outside of the research study, and another 130 patients were withdrawn by their physician, for a total of 37% of the final study population that was included in the primary analysis for this study [4]. Unfortunately, many of the published clinical trials that guide our clinical practice do not specify the number of patients who either were not screened or were ‘partially’ screened and were not enrolled because their health-care provider declined to consider them for inclusion in the research study. The studies of targeted temperature management at 33°C versus 36°C after cardiac arrest [3] and the recombinant activated protein C for adults with septic shock [5], for example, do not clearly specify whether any of the patients were declined for screening and thus for enrollment by their respective health-care providers.

Statistical analyses and regression models would not be able to adjust for such a bias. Sensitivity analyses may shed some light on the characteristics of the excluded patients and how they compare or contrast with the enrolled patient population, but they too cannot convincingly answer the original research question. After all, systematic reviews and meta-analyses may not be at the top of the study design hierarchy, and such analyses may need to be further graded. It might be time to figure out a ‘weighing’ mechanism through which randomized controlled clinical trials would be ‘weighed’ differently based on the intervention’s commercial market availability at the time of the trial. Those trials that were carried out prior to market availability should be given more ‘weight’ than those carried out subsequent to the intervention’s market availability.

In conclusion, the fact that many study interventions are available to clinicians and patients who do not necessarily share the equipoise of the study investigators is potentially a major source of bias that needs to be adequately addressed in every such clinical trial. Every effort must be made to account for such an availability bias, although full accounting is admittedly not possible. Extensive and properly carried out and thought-through sensitivity analyses may help to extrapolate the study findings to those who were excluded from trials, who in many cases are the bulk of the patients considered for that specific intervention in the real clinical non-experimental world.

### Abbreviations

ARDS: Acute respiratory distress syndrome; ARDSnet: Acute respiratory distress syndrome clinical trials network; FACTT: Fluid and catheter treatment trial; PAC: Pulmonary artery catheter.

### Competing interests

The author declares that he has no competing interests.
